# Avasimibe Dampens Cholangiocarcinoma Progression by Inhibiting FoxM1-AKR1C1 Signaling

**DOI:** 10.3389/fonc.2021.677678

**Published:** 2021-05-28

**Authors:** Yunshu Gao, Dongyun Xu, Hongwei Li, Jiahua Xu, Yating Pan, Xinyi Liao, Jianxin Qian, Yi Hu, Guanzhen Yu

**Affiliations:** ^1^ Department of Oncology, People’s Liberation Army General Hospital, Beijing, China; ^2^ Department of Oncology, The 71st Group Army Hospital of People’s Liberation Army, The Affiliated Huaihai Hospital of Xuzhou Medical University, Xuzhou, China; ^3^ Department of Oncology, Longhua Hospital Affiliated to Shanghai University of Traditional Chinese Medicine, Shanghai, China; ^4^ Precision Medical Center laboratory, The First Affiliated Hospital of Wenzhou Medical University, The First Affiliated Hospital of Wenzhou Medical University, Wenzhou, China

**Keywords:** cholangiocarcinoma, FoxM1, AKR1C1, avasimibe, progression

## Abstract

Avasimibe is a bioavailable acetyl-CoA acetyltransferase (ACAT) inhibitor and shows a good antitumor effect in various human solid tumors, but its therapeutic value in cholangiocarcinoma (CCA) and underlying mechanisms are largely unknown. In the study, we proved that avasimibe retard cell proliferation and tumor growth of CCAs and identified FoxM1/AKR1C1 axis as the potential novel targets of avasimibe. Aldo-keto reductase 1 family member C1 (AKR1C1) is gradually increased along with the disease progression and highly expressed in human CCAs. From survival analysis, AKR1C1 could be a vital predictor of tumor recurrence and prognostic factor. Enforced Forkhead box protein M1 (FoxM1) expression results in the upregulation of AKR1C1, whereas silencing FoxM1 do the opposite. FoxM1 directly binds to promoter of AKR1C1 and triggers its transcription, while FoxM1-binding site mutation decreases AKR1C1 promoter activity. Moreover, over-expressing exogenous FoxM1 reverses the growth retardation of CCA cells induced by avasimibe administration, while silencing AKR1C1 in FoxM1-overexpressing again retard cell growth. Furthermore, FoxM1 expression significantly correlates with the AKR1C1 expression in human CCA specimens. Our study demonstrates a novel positive regulatory between FoxM1 and AKR1C1 contributing cell growth and tumor progression of CCA and avasimibe may be an alternative therapeutic option for CCA by targeting this FoxM1/AKR1C1 signaling pathway.

## Introduction

Cholangiocarcinoma (CCA) is an aggressive cancer in bile ducts with a high frequency of recurrence and an extremely poor prognosis. In the past decade, the morbidity and mortality of CCA were on the rise worldwide (1–3). Numerous risk factors induce CCA, including primary sclerosing cholangitis, parasitic infections and choledochal cysts (4). Surgical resection is still the mainstay of potentially curative for CCA patients diagnosed at early-stage disease. For patients with advanced-stage or unresectable CCA, the effectiveness of systemic therapies is limited, and the median overall survival is less than one year (5). Recent progress in molecular genetics provide one avenue to develop pharmacological inhibitors of pathologic mutations. However, whether patients with advanced CCA could obtain a benefit from genetic profiling and classifications underlying CCA tumorigenesis, as well as screening more effective therapeutic strategies, including conventional chemotherapy, radiotherapy, targeted therapies, and immunotherapy remain largely unclarified.

Avasimibe, a bioavailable acetyl-CoA acetyltransferase (ACAT) inhibitor, has long been regarded as a promising antihyperlipidemic and antiathero-sclerotic drugs (6, 7). Avasimibe can directly reduce atherosclerotic activity, cholesterol level, macrophage infiltration, and the expression and activity of matrix metalloproteinase (7). Recently, increasing evidences showed antitumor effects of avasimibe on a variety of human solid tumors, including melanoma, hepatocellular carcinoma, and osteosarcoma (8–10). These reports demonstrate that avasimibe markedly inhibited tumor growth *in vivo* by targeting the downstream targets, such as Sterol O-Acyltransferase 1 (SOAT1) (8) and Acetyl-CoA Acetyltransferase 1 (ACAT-1) (11). To deepen the understanding of Avasimibe, our group focused on the discovery of new targets of Avasimibe. Forkhead Box M1 (FoxM1) is a member of Forkhead transcription factors family, working as an oncogene in human malignant tumors (12). Aldo-keto reductase 1 family member C1 (AKR1C1) has been well-known to be involved in carcinogen metabolism. AKR1C1 expression is related to development and metastasis of many types of cancer (13–16). Our recent study suggested that AKR1C1 is a novel target of FoxM1 and FoxM1/AKR1C1 signaling is inhibited by avasimibe at osteosarcoma (9). However, whether avasimibe has the same therapeutic effectiveness on cholangiocarcinoma is unknown. Moreover, the mechanism underlying avasimibe-inhibited tumorigenesis is remains poorly understood.

We aim to assess the antitumor effect of avasimibe on cholangiocarcinoma and to explore its potential mechanism. Our results showed the inhibitory effect of avasimibe on CCA *in vivo* and *in vitro* and demonstrated that avasimibe targets FoxM1/AKR1C1 signaling, an essential pathway in tumorigenesis and cancer progression. Our finding may promote the clinical application of avasimibe in the treatment for CCA.

## Materials and Methods

### Cell Culture

CCA cell lines RBE and QBC939 were preserved in our lab. CCA cells were cultured in Dulbecco’s modified Eagle’s medium (DMEM) supplemented with 10% fetal bovine serum (FBS), 1% penicillin/streptomycin, and 1% glutaMAX (Invitrogen). Recombinant avasimibe was purchased from Selleck (S2187) for *in vitro* study and Shanghai super LAN chemical technology center for *in vivo* study with the final treatment concentration of 30 mg/kg.

### Tissues of Patients

Human hilar cholangiocarcinoma tissue microarray preserved in our lab (17) and 49 patients with no preoperative chemotherapy or radiation therapy were enrolled in this study. Of the 49 patients, 35 (71.4%) are male patients and 14 patients (28.6%) are female. Of these patients, 20 (40.8%) had TNM stage I/II tumors, and 29 (59.2%) had TNM stage III/IV tumors. All patients had clinical follow-up, with a median follow-up of 23 months (1-59 months). The institutional review boards of Eastern Hepatobiliary Hospital approved the use of the tissues and clinical information in this study.

### Animal Models

Abdominal cavity tumor xenograft model was used to evaluate the therapeutic effect of Awasimibe. QBC939 cells (1×10^6^) were trypsinized and resuspended in PBS. Then, cells were injected into 6-week-old Balb/c nude mice (n=13). After one week implantation, mice were divided into control group (n=6) and an avasimibe-treated group (n=7). The avaximide treatment group was given avaximide by gavage for 21 days. All animals were sacrificed on the 22nd day and the tumor weight was determined. All experiments were based on the National Institutes of Health Guide for the Care and Use of Laboratory Animals and were approved by the Animal Ethics Committee of the Second Military Medical University.

### cDNA Array

RBE cells were treated with 20 µM avasimibe. After 24 and 48 hours, cells were collected and extracted total mRNA for cDNA microarray analysis. The Affymetrix human genome U219 array was used to analyze the differential gene expression. Gene cluster analysis was used to determine the genes and pathways with the most significant changes (9).

### Plasmids and Transfections

Human AKR1C1 were cloned into pCDH-Flag vectors. The pGIPZ shNT was generated with the control oligonucleotide 5’-GCTTCTAACACCGGAGGTCTT-3’. pGIPZ AKR1C1 shRNA was generated with 5’ - AGAAAGGAAAGACAATAATTT-3’ oligonucleotide. The pcDNA3.1-FoxM1 and FoxM1-shRNA plasmids were gifts from Prof. Sunyun Huang (MD Anderson Cancer Center, Houston, TX, USA) (18). PCR-amplified AKR1C1 promoter sequence (WT), FoxM1-binding motifs 1 deletion mutant (Mut1), FoxM1-binding motifs 2 deletion mutant (Mut2), FoxM1-binding motifs 1 deletion mutant (Mut3), FoxM1-binding motifs 1 deletion mutant (Mut4) and FoxM1-binding motifs 1 deletion mutant (Mut5) were cloned into pGL3-promoter vectors.

Cells were seeded and then transfected with the indicated plasmids using Polyjet In Vitro DNA transfection Reagent (SignaGen) according to the manufacturer’s instructions.

### Cell Proliferation

CCA cells were plated in 96-well plates (1×10^4^/well) and then treated with avasimibe at concentrations of 0 μM, 10 μM, and 20 μM. For stably transfected CCA cells with shAKR1C1, 5000 CCA cells per well were seeded in 96-well plates. At the indicated times, the cell viability was measured by a CCK8 assay (Dojindo). These experiments were performed in triplicate.

### Immunohistochemistry

4 μm-thick paraffin-embedded sections of CCA samples and tumors in mice were prepared and processed for immunohistochemistry. FoxM1 (sc-500, Santa Cruz Biotechnology), PCNA (sc-500, Maixin-Bio, Fuzhou, China), and AKR1C1 (PB1091, Boster Biological Technology, Wuhan, China) antibodies were used for IHC analysis. A streptavidin-biotin kit (#KIT-9720; Maixin-Bio) was used to visualize antibody binding to the tissues. Two individuals (G. Y. and Y. C.) independently evaluated all these samples. The final results generated using a semiquantitative scoring system as previously described (17, 19). Briefly, the mean percentage of tumor cells positive for indicated marker(s) was calculated in five areas of a given sample at a magnification of × 400 and scored from 0 to 1 (0-100%). The intensity of immunostaining was scored as 0 for negative, 1 for weak, 2 for moderate, and 3 for strong. Theoretically, a weighted score was generated for each case, ranging from 0 (0% of cells stained) to 300 (100% of cells stained at 3+ intensity). The cutoff points were based on the scores: negative, 0; weak, <75; moderate, 76–150; and strong, >151. We defined the score <75 as low expression and ≥75 as high expression. Their correlations determined by Pearson’s correlation test.

### Quantitative Real-Time PCR

Total RNA was extracted with Trizol reagent (Life technologies). cDNA was reversed by a ReverTra Ace qPCR RT Master Mix Kit (Toyobo) qRT-PCR was performed by Bio-Rad PCR Thermal Cyclers. Data were normalized to β-actin for each experiment. The following primer pairs were used for quantitative real-time PCR: AKR1C1, 5’-TATGCGCCTGCAGAGGTTC-3’ (forward) and 5’-TCAATATGGCGGAAGCCAGC-3’(reverse); FoxM1, 5’- GGAGCAGCGACAGGTTAAGG-3’ (forward) and 5’-TCAATATGGCGGAAGCCAGC-3’(reverse); β-actin, 5’-CATGTACGTTGCTATCCAGGC-3’ (forward) and 5’-CTCCTTAATGTCACGCACGAT-3’ (reverse).

### Luciferase Reporter Assay

AKR1C1 promoter reporter plasmids (- 500bp), mutant PDGF-A promoter reporter plasmids (by deleting FoxM1-binding site, respectively), Renilla luciferase (pRL-TK) vector plasmid, were transfected. After 48 hours, cells were harvested and tested with the Dual-Glo luciferase reporter assay system (Promega). All experiments were performed in triplicate times.

### Chromatin Immunoprecipitation (ChIP) Assay

ChIP assays were performed through SimpleChIP Enzymatic Chromatin IP kit (Cell Signaling Technology) according to its manufacturer’s instructions. The purified DNA fragments were subjected to semiquantitative PCR analysis using site-specific primers: h-AKR1C1-CHIP-1-F: 5’-CCAAAGTCCAAAAGCTGTTAATAAGAAATCTTC-3’; h-AKR1C1-CHIP-1-R: 5’-TGCATTACTTTTTTCATCAGCAAATTTATTGTTCC-3’; h-AKR1C1-CHIP-2-F: 5’-GAGGTTTCTGTATTCTTATGTAAAGTCACAATTTGT-3’; h-AKR1C1-CHIP-2-R: 5’-TGCATCCAGTTCAACCGTTTCTTAC-3’.

### Western Blotting

All treated and untreated cells were lysed with RIPA and resolved in SDS-PAGE and transferred to PVDF membranes (Bio-Rad Laboratories, Hercules, CA, USA). The membranes were probed with antibodies, and then detected with an enhanced chemiluminescence (ECL) kit (Santa Cruz, CA, USA).

### Statistics

All analyses were performed using SPSS and GraphPad Prism (version 5.0) software. Categorical data were analyzed using χ^2^ tests. Differences between groups were determined using a two-tailed Student’s t-test. For survival rate analysis, the Kaplan-Meier method was used to estimate survival rates, and the log-rank and Wilcoxon rank sum tests were performed to assess survival differences between groups. A *p* value less than 0.05 was considered statistically significant.

## Results

### Avasimibe Administration Inhibited Cell Proliferation and Tumor Growth

**Figure 1:**
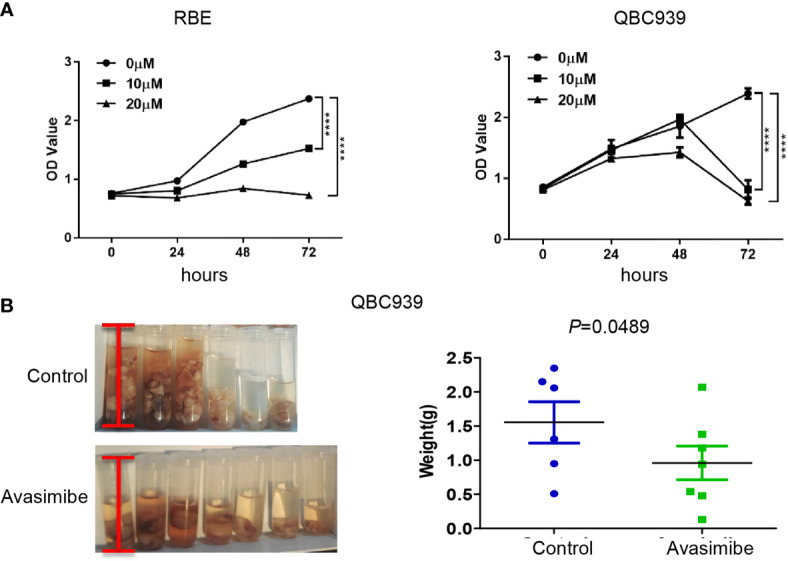
The inhibitory effect of avasimibe treatment on cell proliferation and tumor growth of cholangiocarcinoma. **(A)** RBE and QBC939 cells were treated with avasimibe at indicated concentrations (0μM, 10μM, and 20μM) and cell proliferation was measured by CCK8 assay at 24h, 48h, and 72h. ****P < 0.0001. **(B)** QBC939 cells (1×106 per mouse) were injected into the abdominal cavity of 6-week-old female Balb/c nude mice. Mice were given avasimibe (n=7) or regular water (n=6) by oral gavage for 21 days. The tumor volume and tumor weight were examined at 22 days. CCK8, Cell Counting Kit-8.

It was demonstrated that administration of avasimibe inhibited the CAA cell proliferation in dose-dependent and time-dependent manners *in vitro* (**Figure 1A**). *In vivo* assay demonstrated that avasimibe administration resulted in a remarkable reduction of tumor volume and tumor weight of QBC 939 cells (**Figure 1B**).

### Avasimibe Inhibits the Expression of AKR1C1 and Cell Division

**Figure 2:**
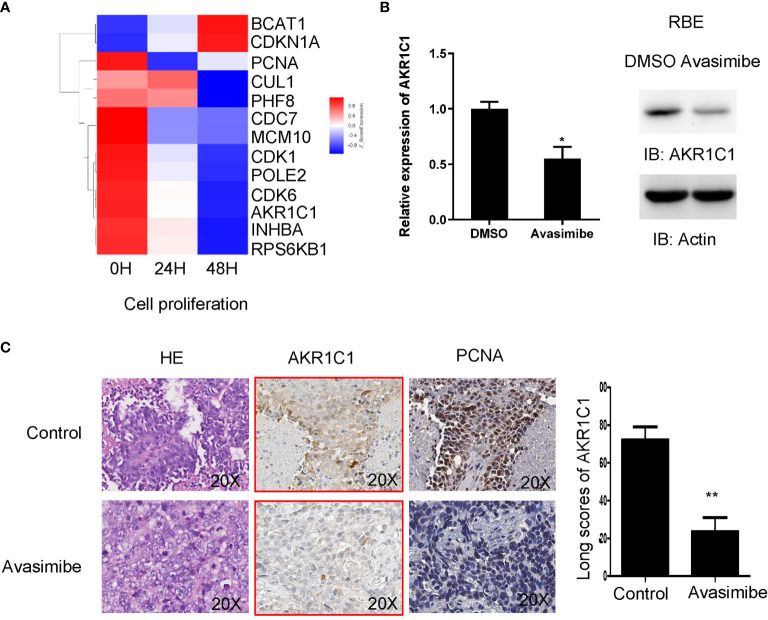
cDNA array analysis identified AKR1C1 as a potential target of avasimibe. **(A)** RBE cells were treated with avasimibe at the concentration of 20 µM for 24 and 48 hours and subjected to cDNA array analysis. Cluster of changed genes in cell proliferation was presented. **(B)** The level of AKR1C1 mRNA and protein was detected by RT-PCR and western blotting in RBE cells when treated with avasimibe (20µM) for 48 hours. **(C)**The expression of AKR1C1 and PCNA was detected by IHC on the resected xenografts. IHC, ×200. *P < 0.05, **P < 0.01. ‘long scores of AKR1C1’ means AKR1C1 staining score. IHC, immunohistochemistry.

Given the importance of avasimibe in inhibiting cell proliferation and tumor progression, cDNA array was carried out to assess the transcriptional effects of avasimibe in RBE cells in a time-dependent manner. By comparing the gene expression patterns after avasimibe treatment to that at base line, a total of 5,966 genes were significantly altered at 24 hours, while 11,055 genes were altered at 48 hours (**Figure S1A**). Gene ontology analysis demonstrated that the majority of the most valuable clusters were associated with cell proliferation (**Figures S1B, C**), and the potentially significant candidate genes were shown in **Figure 2A**. Among these genes, AKR1C1 was one remarkably decreased gene along with the administration of avasimibe, which was validated by both RT-PCR and immunoblotting assays (**Figure 2B**). Further immunohistochemical analysis showed that avasimibe treatment reduced the expression of AKR1C1 and PCNA, another target of avasimibe revealed by cDNA array, in the xenograft samples (**Figure 2C**).

### The Oncogenic Role of AKR1C1 in Human Cholangiocarcinoma

**Figure 3:**
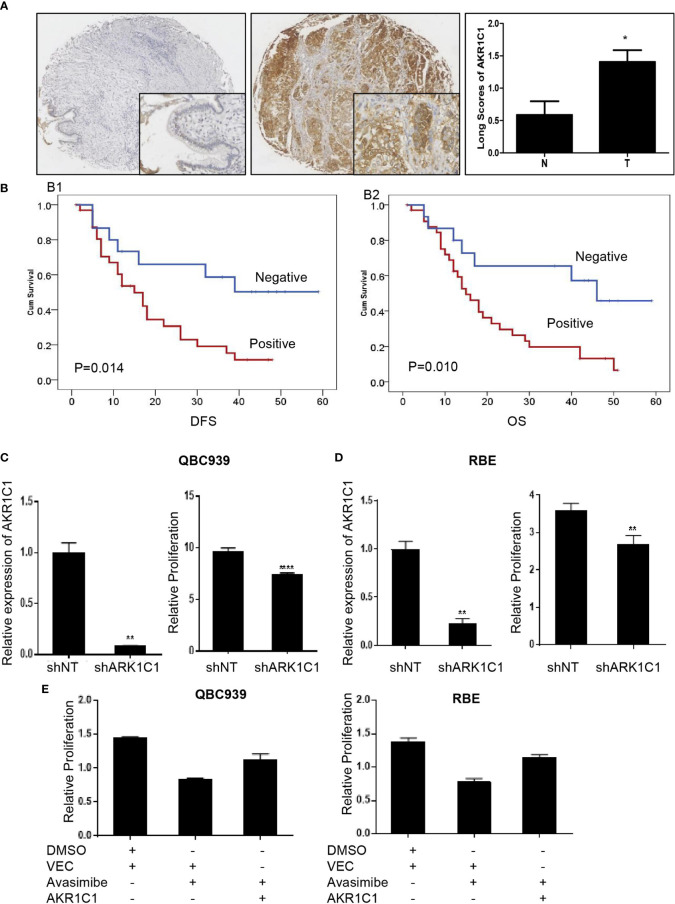
The oncogenic role of AKR1C1 in cholangiocarcinoma. **(A)** Representative images of negative staining of AKR1C1 in noncancerous tissues (N) and high expression of AKR1C1 in CCA (T). IHC,×200 for small pictures and×40 for large pictures. *P < 0.05. **(B)** Patients with AKR1C1 expression had a shorter time to recurrence (B1) and a worse overall survival (B2) than those without AKR1C1 expression. **(C, D)** QBC939 **(C)** and RBE **(D)** cells were transfected with AKR1C1-shRNA for 48 hours and the level of AKR1C1 mRNA was detected by RT-PCR. CCK8 assay was used to measure the cell viability of both cell lines. **P < 0.01, ****P < 0.0001. **(E)** QBC939 and RBE cells were treated with avasimibe with or without exogenous AKR1C1 plasmid for 48 hours and CCK8 was used to detect the changes of cell viability. IHC, immunohistochemistry; CCK8, Cell Counting Kit-8; Vec, vector.

AKR1C1 was weakly or absently observed in the cytoplasm of normal bile duct cells, but dramatically increased in tumor cells (**Figure 3A**). Overall, AKR1C1 was overexpressed in 69.4% (34/49) cases, and the high expression of AKR1C1 is related to regional lymph node metastasis and nerve invasion (**Table 1**). The prognosis of patients with AKR1C1 positive tumors were significantly lower than that of patients without AKR1C1 expression. (15 mon vs 38 mon, P=0.014) (**Figure 3B1**). In the multivariate analysis, T stage and N stage were independent predictive factors (**Table 2**). As for the overall survival duration, T stage, regional lymph node metastasis, positive margin, and AKR1C1 expression were significant prognostic factors. Patients with AKR1C1-positive tumors had a significantly poor outcome than those without AKR1C1 expression (13 mon vs 50 mon, P<0.001) (**Figure 3B2**). In the multivariate Cox model, T stage expression was an independent prognostic factor (**Table 3**).

**Table 1:** Correlation between AKR1C1 expression and clinicopathological factors.

Parameters	AKR1C1		
	N	n (%)	*P*
Median age		55y (31-79)	
Gender			
Male	35	22 (62.9)	0.117
Female	14	12 (88.7)	
Nerve invasion			
No	26	14 (53.8)	0.012
Yes	23	20 (87.0)	
T stage			
T1-3	7	3 (42.9)	0.100
T4	42	31 (73.8)	
N stage			
No	16	7 (43.8)	0.007
Yes	33	27 (81.8)	
Differentiation			
High/moderate	37	24 (64.9)	0.228
poor/un-differentiated	12	10 (83.3)	
Disease stage			
I/II	20	11 (55.0)	0.070
III/IV	29	23 (79.3)	

**Table 2:** Univariate and multivariate analysis of time to progression in 49 patients with hilar cholangiocarcinoma according to clinicopathologic factors and AKR1C1 overexpression.

Clinicopathological factor	Case (n)	TTP (mo)	Univariate		Multivariate		
			χ2	*P*	χ2	*P*	*HR (95%CI)*
T stage							
T1-3	7	>52	9.437	0.002	6.412	0.011	0.071(0.009-0.551)
T4	41	16					
N stage							
No	16	39	4.759	0.029	4.261	0.039	0.401(0.168-0.955)
Yes	32	15					
Differentiation							
High/moderate	36	26	3.450	0.063		NA	
poor/un-differentiated	12	11					
Positive margin							
No	23	37	3.983	0.046		NA	
Yes	25	17					
AKR1C1							
Negative	15	38	8.068	0.014		NA	
Positive	33	15					

**Table 3:** Univariate and multivariate analysis of overall survival in 49 patients with hilar cholangiocarcinoma according to clinicopathological factors and AKR1C1 overexpression.

Clinicopathological factor	Case (n)	OS(mo)	Univariate analysis		Multivariate analysis		
			χ2	*P*	χ2	*P*	*HR(95%CI)*
T stage							
T1-3	7	50	7.559	0.006	6.172	0.013	0.153(0.035-0.672)
T4	42	16					
N stage							
No	16	42	5.602	0.018	3.343	0.067	0.459(0.199-1.058)
Yes	33	14					
Differentiation							
High/moderate	37	23	3.640	0.056		NA	
poor/un-differentiated	12	16					
Positive margin							
No	23	26	4.096	0.043		NA	
Yes	26	16					
AKR1C1							
Negative	19	50	24.923	<0.001		NA	
Positive	30	13					

We next examined the function of AKR1C1 in CCA cells by silencing AKR1C1 *in vitro*. As expected, knockdown of AKR1C1 resulted in significant inhibition of cell proliferation of CCA cells (**Figures 3C, D**). Moreover, enforced expression of AKR1C1 could rescue the inhibitory effect of avasimibe on CCA cells (**Figure 3E**).

### Avasimibe Inhibits AKR1C1 Expression Through FoxM1

**Figure 4:**
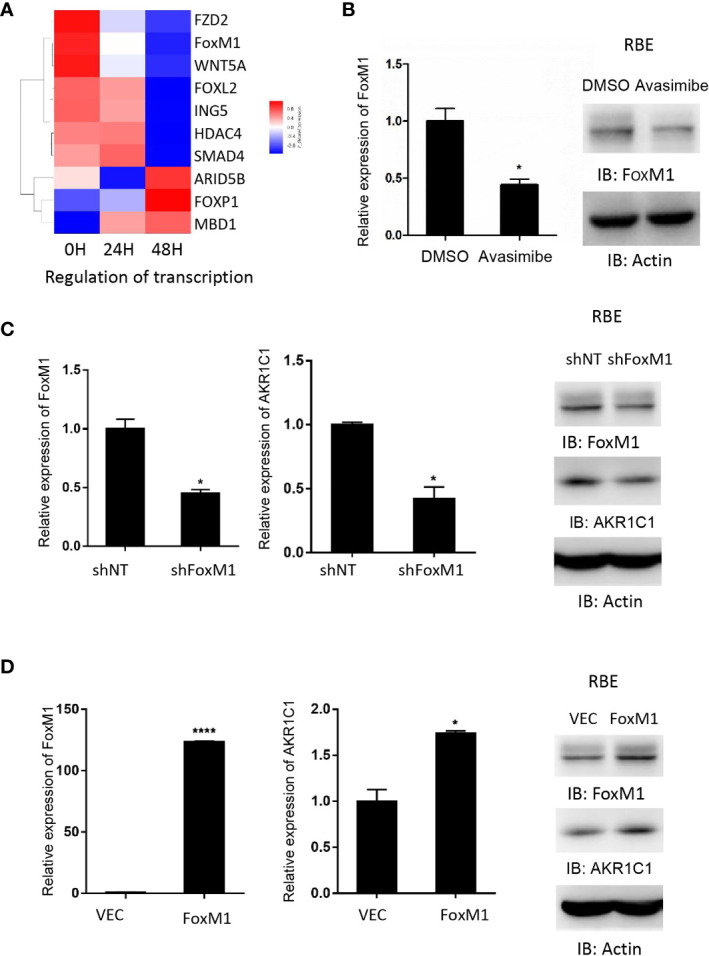
AKR1C1 is regulated by FoxM1 in cholangiocarcinoma. **(A)** RBE cells were treated with avasimibe (20 µM) for 24 and 48 hours and subjected to cDNA array analysis. Cluster of changed genes in regulation of transcription was presented. **(B)** The level of FoxM1 mRNA and protein was detected by RT-PCR and western blotting in RBE cells when treated with avasimibe (20µM) for 48 hours. **(C)** RBE cells were transfected with FoxM1-shRNA for 48 hours and the levels of FoxM1 and AKR1C1 mRNA and proteins were detected by RT-PCR and western blotting. **(D)** RBE cells were transfected with FoxM1 plasmid for 48 hours and the levels of FoxM1 and AKR1C1 mRNA and proteins were detected by RT-PCR and western blotting. *P < 0.05, ****P < 0.0001.

Gene ontology analysis demonstrated that FoxM1, a transcription factor, was decreased by avasimibe treatment (**Figure 4A**). This result was also validated by both RT-PCR and western blotting assays (**Figure 4B**). To investigate the relationship between FoxM1 and AKR1C1, we firstly detected the expression level of FoxM1 and AKR1C1 mRNA in established stably FoxM1 or shFoxM1-transfected CCA cells. Silencing FoxM1 in RBE cells led to decreased expression of endogenous AKR1C1 (**Figure 4C**), while overexpression of FoxM1 resulted in an increase in endogenous AKR1C1 expression (**Figure 4D**). These results indicated that FoxM1 is partially responsible for the induction of AKR1C1 expression.

### FoxM1 Directly Regulates the Promoter Activity of AKR1C1

**Figure 5:**
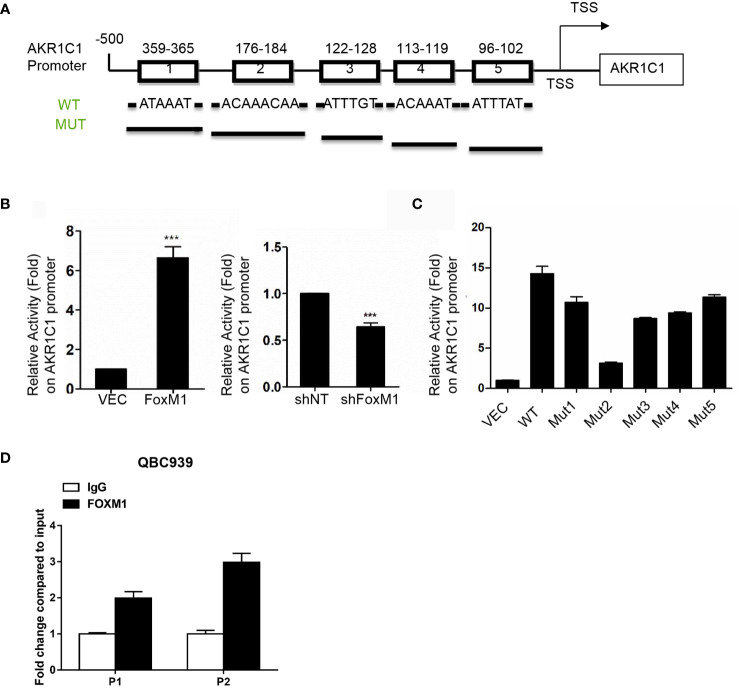
AKR1C1 is a direct transcriptional target of FoxM1. **(A)** Diagram shows the sequence and position of five putative FoxM1-binding elements in the AKR1C1 promoter. TSS, transcriptional start site; WT, wild type; Mut, mutant type. **(B)** Left panel, RBE cells were cotransfected with the AKR1C1 promoter reporter, pRL-TK, and pcDNA3.1-FoxM1 or pcDNA 3.1; right panel, RBE cells were cotransfected with the AKR1C1 promoter reporter, pRL-TK, and FoxM1-shRNA or shcontrol (50 nM). 36 hours after transfection, the cells were collected, and the relative AKR1C1 promoter activities were measured. The assay was repeated three times independently. ***P < 0.001. **(C)** Reporter plasmids harboring the wild-type AKR1C1 promoter or the corresponding mutant promoter in the FoxM1-binding sites were transfected into RBE cells, and the relative promoter activities were measured as above. **(D)** The chromatin immunoprecipitation (ChIP) assay results show the *in vivo* binding of FoxM1 to the AKR1C1 promoter. QBC939 cell lysis was immunoprecipitated using an anti-FoxM1 antibody or immunoglobulin G The resulting samples were subjected to RT-PCR using the site-specific primers.

We further aimed to investigate whether FoxM1 could bind to AKR1C1 promoter region to regulate is expression. We scanned ~500bp promoter regions of the AKR1C1 gene containing the FoxM1 binding consensus sequence. Five FoxM1 putative binding sites (-780 to -774 bp and-948 to -942 bp) were found in the promoter region (**Figure 5A**). Next, we cloned DNA sequence containing the five potential FoxM1 binding sites and constructed a PGL3-AKR1C1 promoter plasmid. When we cotransfected PGL3-AKR1C1 promoter with pcDNA3.1-FoxM1B or pcDNA3.1 in RBE cells, the activity of PGL3-AKR1C1-promoter was upregulated in FoxM1B-transfected cells (**Figure 5B**). Meanwhile, knockdown of FoxM1 in RBE cells inhibited the activity of PGL3-AKR1C1-promoter (**Figure 5B**). To further confirm this result, we mutated the FoxM1 DNA binding sites and found that the activity of PGL3-ADAM17-mutated promoter was decreased (**Figure 5C**). The second FoxM1 DNA binding site was the most important site responsible for FoxM1-AKR1C1 interaction. Moreover, ChIP assays using antibodies specific against either FoxM1 or IgG (control) showed that FoxM1 is directly bound to the endogenous AKR1C1 promoter region on both sites compared with IgG control (**Figure 5D**). Together, these data indicated that AKR1C1 is a direct transcriptional target of FoxM1.

### Avasimibe Targets FoxM1-AKR1C1 Signaling in CCA

**Figure 6:**
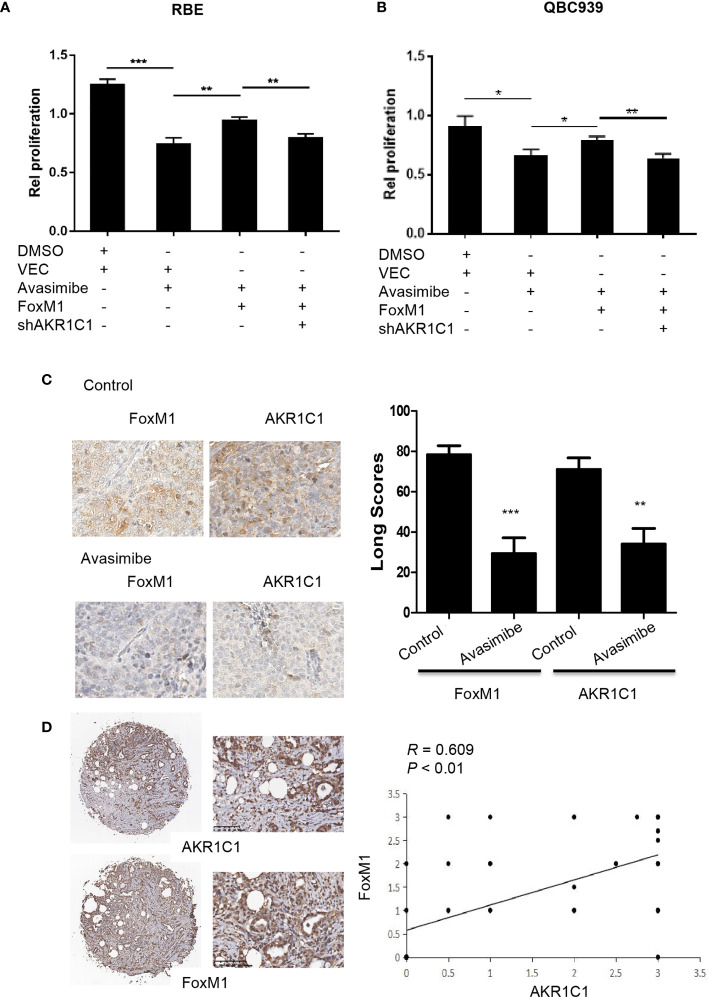
Avasimibe inhibits cholangiocarcinoma cells proliferation *via* targeting AKR1C1 and FoxM1. **(A, B)** RBE cells **(A)** and QBC939 cells **(B)** were treated with avasimibe or DMSO, then transfected with FoxM1 or control vector, along with the transfection with AKR1C1 shRNA or shNT. 48 h after transfection, cell viability was analyzed by CCK8 assay. Data are from three independent assays. *P < 0.05, **P < 0.01, ***P < 0.001. **(C)** Left panel, the expression of FoxM1 and AKR1C1 was detected by IHC on the resected xenografts. IHC, ×400. Right panel, diagram showing the different expression of FoxM1 and AKR1C1 in these samples when treated with avasimibe. **(D)** The representative images of FoxM1 and AKR1C1 expressions and their correlations determined by Spearman’s correlation test. r, Spearman correlation coefficient; IHC, ×40 or ×200.

To investigate whether FoxM1 could regulate the effects of avasimibe on cholangiocarcinoma cells proliferation, we overexpressed FoxM1 in RBE cells and QBC9393 cells before avasimibe treatment, and we found FoxM1 overexpression could counteract the effects of avasimibe on cholangiocarcinoma cells proliferation (**Figures 6A, B**). Nevertheless, if we knockdown AKR1C1 at the same time, the effects of FoxM1 will be neutralized (**Figures 6A, B**).

In consistent with the above study, IHC showed that FoxM1 and AKR1C1 were highly expressed in xenografts in nude mice compared with those treated with avasimibe (**Figure 6C**). We further detected the levels of both FoxM1 and AKR1C1 in human CCA samples. The expression profile of FoxM1 was consistent with the trend of AKR1C1 expression. FoxM1 was expressed in 33 samples, while AKR1C1 was expressed in 69.39% samples (49). FoxM1 overexpression correlated with AKR1C1 overexpression (**Figure 6D**).

## Discussion

In the present study, we demonstrate strong evidence supporting the potential therapeutic role of avasimibe in treating hilar cholangiocarcinoma. We proved that avasimibe alone inhibited cell viability and tumor growth of human CCA cells by targeting the FoxM1/AKR1C1 signaling pathway. Specifically, FoxM1 regulates AKR1C1 expression by directly binding to its promoter. AKR1C1 was a promising predictor for tumor recurrence and overall survival and correlated significantly with FoxM1 expression in CCAs. The FoxM1/AKR1C1 pathway might be a key mechanism for human CCAs tumorigenesis and a potential therapeutic target for avasimibe.

Avasimibe potentiated the antitumor effect of an anti-PD-1 antibody (10) and blocked cholesterol esterification leading to apoptosis and suppression of proliferation of several cancer cells (20). However, whether avasimibe can prevent the progression of CCA is unclear. Here, we proved for the first time that avasimibe can inhibit the proliferation of CCA cells *in vitro* and tumor growth *in vivo*, indicating its potential application in therapeutic strategies. Avasimibe was originally designed as an ACAT inhibitor (CI-1011) (21) and ACAT is an intracellular membrane-bound enzyme involved in cellular cholesterol homeostasis. Upregulated expression of ACAT has been observed in many types of cancer, making cholesterol metabolism as a potential target for cancer treatment (22), including ACAT1 and ACAT2 (23). Our cDNA array analysis showed that ACAT2, not ACAT1, was downregulated in CCA cells after the administration of avasimibe. These results are inconsistent with previous reports (8, 10) suggesting that the anti-tumor effect of avasimibe must have more complicated mechanisms. In consistent with our previous research in osteosarcoma (9), avasimibe has a direct killing effect on bile duct cancer cells revealed by gene cluster analysis and altered genes related to cell proliferation, including CDK1, CDK6, cyclin B1, FoxM1, and, especially, Ki67 and PCNA, two essential biomarkers representing the ability of cell proliferation. Taken together, avasimibe will be an efficient therapy in the treatment of CCA.

Although designed to target cholesterol metabolism, avasimibe was found to block various members of AKR1 family in human steroid metabolism (24), including AKR1B1, AKR1B10, and AKR1C1. Aldo-keto reductases are well known as metabolic enzymes of carbonyls, but recent data indicates that multiple embers in AKR families are involved in the development of various human solid tumors (25). For example, dysregulated expression of AKR1B10 in hepatocellular Carcinoma (26), breast cancer (27), and colorectal cancer (28) make AKR1B10 inhibitors as potential drugs for cancer treatment (29). AKR1C1, another member of the AKR1 family, has been well-known to be involved in carcinogen metabolism. AKR1C1 expression is related to development and metastasis of many types of cancer (13–16). In present study, we validated that AKR1C1 was an effective target of avasimibe and expression of AKR1C1 was closely correlated with the metastatic potential of CCA. Moreover, in consistent with previous studies (9, 16), AKR1C1 expression in carcinoma cells correlated positively with DFS and OS of CCA. Targeting AKR1C1 either by avasimibe or by AKR1C1-shRNA significantly repressed the proliferation of CCA cells. Taken together, AKR1C1 is involved in the development and progression of CCA, making it a potential new anti-cancer target.

The critical roles of catalytic-dependent and catalytic-independent function of AKR1C1 in regulating biological events have been well-summarized (30). AKR1C1 degrades Progesterone to its metabolite 20α-DHP, influencing progesterone metabolism associated with breast cancer (31). AKR1C1 directly activates STAT3 by facilitating its phosphorylation (13). However, the detailed information of transcriptional activation of AKR1C1 is not well-studied. Our previous study showed that FoxM1 was decreased by avasimibe treatment and was a potential activator of AKR1C1 (9). However, the mechanisms underlying FoxM1 activating AKR1C1 are largely unknown. Here, we not only confirmed that the administration reduced AKR1C1 expression, but also showed that FoxM1 could positively regulate AKR1C1 expression in CCA cells. Further study demonstrates that AKR1C1 is a directly transcriptionally regulated by FoxM1 in CCA. Therefore, this study disclosed a novel mechanism for AKR1C1 activation in the progression of CCA.

The inhibitory effect of avasimibe on FoxM1/AKR1C1 signaling pathway is irrefutable evident. The question is whether avasimibe directly targets either FoxM1 or AKR1C1, or whether it firstly acts on FoxM1 and then inactivates AKR1C1, leading to retarded growth of CCA cells. In present study, we showed that over-expressing exogenous FoxM1 reversed the growth retardation of CCA cells induced by avasimibe administration. Meanwhile, silencing AKR1C1 in FoxM1-overexpressing again retarded cell growth when administrated with avasimibe. Thus, we proved that decreased expression of AKR1C1 induced by avasimibe was partially through decreased expression of FoxM1 targeted by avasimibe.

In summary, our findings showed the great importance of FoxM1/AKR1C1 signaling pathway in human cholangiocarcinoma. Meanwhile, the FoxM1/AKR1C1 axis in human cancers was the potential target of avasimibe which successfully retarded cell proliferation and tumor growth of CCA. Therefore, our findings suggest that avasimibe can be used in cholangiocarcinoma treatment.

## Data Availability Statement

The original contributions presented in the study are included in the article/**Supplementary Material**. Further inquiries can be directed to the corresponding authors.

## Ethics Statement

The studies involving human participants were reviewed and approved by The Institutional Review Boards of Eastern Hepatobiliary Hospital. The patients/participants provided their written informed consent to participate in this study. The animal study was reviewed and approved by the Animal Ethics Committee of the Second Military Medical University.

## Author Contributions

Conception/Design: JQ, YH, and GY. Collection and/or assembly of data: YG, DX, HL, and YP. Data analysis and interpretation: HL, JX, JQ, and XL. Manuscriptwriting: YH and GY. Final approval of manuscript: JQ, YH, and GY. Funding support: GY and DX. All authors contributed to the article and approved the submitted version.

## Funding

This work was supported by National Natural Science Foundation of China (81972721), Medical Science Research Foundation of People’s Liberation Army (14ZD16) and Research project of Xuzhou Medical University (2018kj15).

## Conflict of Interest

The authors declare that the research was conducted in the absence of any commercial or financial relationships that could be construed as a potential conflict of interest.

## Supplementary Material

Supplementary Figure 1
**(A)** RBE cells were treated with avasimibe for 24 and 48 hours and subjected to cDNA array analysis. Intersection of genes between 24/0 and 48/0 hour. **(B)** Significant GO terms retrieved by clusters of genes involved in the union of A. **(D)** Significant GO terms retrieved by clusters of genes involved in the intersection of A. GO, gene ontology.

## Abbreviations

CCA, Cholangiocarcinoma; IHC, Immunohistochemistry; AKR, Aldo-keto reductase.
