# Supplementary figures and images for: Avasimibe Dampens Cholangiocarcinoma Progression by Inhibiting FoxM1-AKR1C1 Signaling
Source: Front Oncol. 2021 May 28;11:677678. doi: 10.3389/fonc.2021.677678 (PMC8195695; doi:10.3389/fonc.2021.677678)

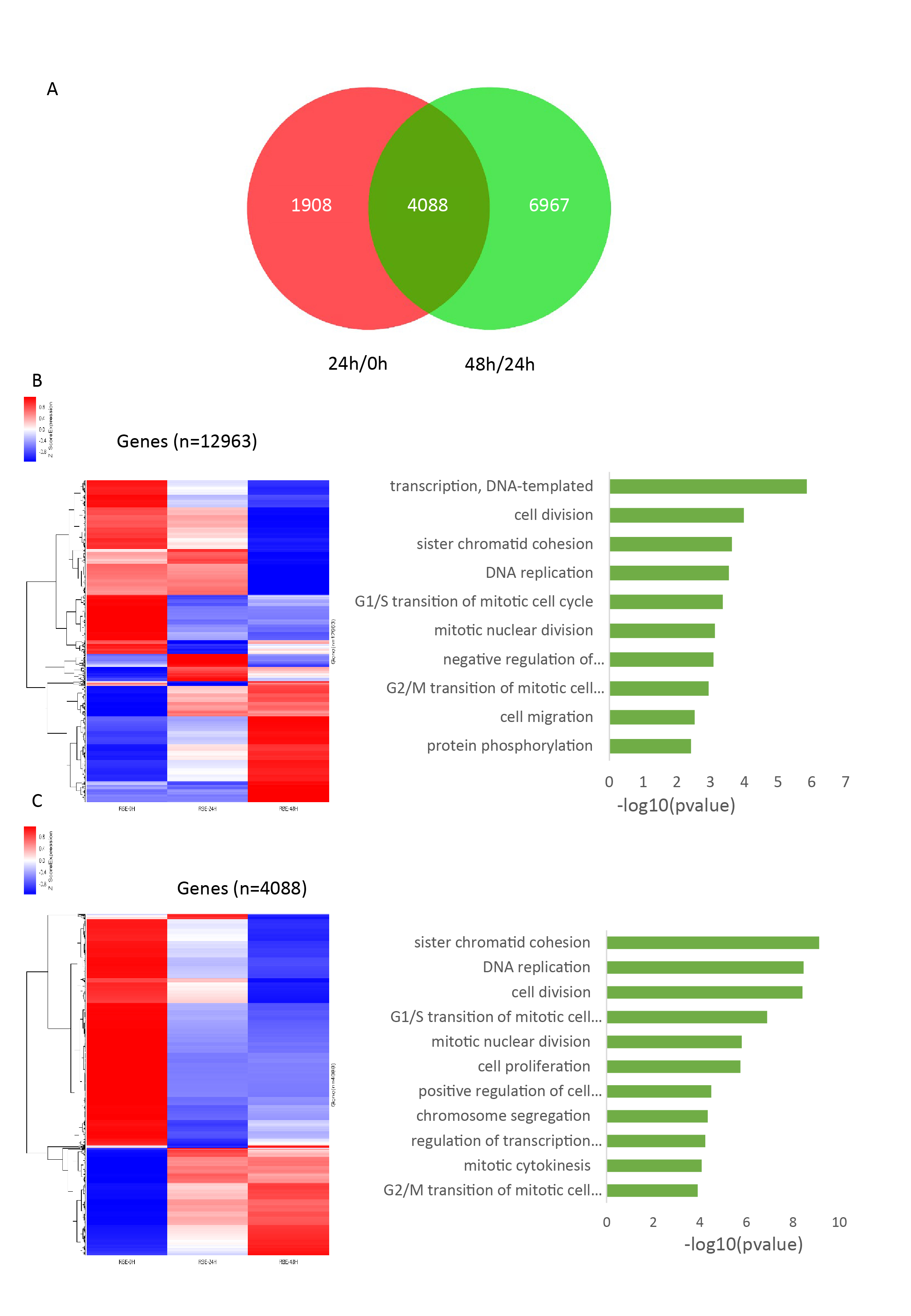

Supplement: Supplementary Figure 1 — (A) RBE cells were treated with avasimibe for 24 and 48 hours and subjected to cDNA array analysis. Intersection of genes between 24/0 and 48/0 hour. (B) Significant GO terms retrieved by clusters of genes involved in the union of A. (D) Significant GO terms retrieved by clusters of genes involved in the intersection of A. GO, gene ontology. [file Image_1.jpeg]
